# Gene polymorphisms and serum levels of sVEGFR-1 in patients with systemic lupus erythematosus

**DOI:** 10.1038/s41598-020-72020-8

**Published:** 2020-09-14

**Authors:** Zhi-Chao Yuan, Wang-Dong Xu, Jia-Min Wang, Qian Wu, Jie Zhou, An-Fang Huang

**Affiliations:** 1 Department of Evidence-Based Medicine, School of Public Health, Southwest Medical University, 1 Xianglin Road, Luzhou, 646000 Sichuan People’s Republic of China; 2 Department of Rheumatology and Immunology, Affiliated Hospital of Southwest Medical University, 25 Taiping Road, Luzhou, 646000 Sichuan People’s Republic of China

**Keywords:** Genetics, Immunology

## Abstract

Correlation between soluble vascular endothelial growth factor receptor-1 (sVEGFR-1) concentration, VEGFR1 gene polymorphisms and systemic lupus erythematosus (SLE) risk remains unclear. The present case–control study comprised 254 SLE patients, 385 other rheumatic diseases patients and 390 healthy controls. Serum levels of sVEGFR-1 were detected by enzyme-linked immunosorbent assay. Seven VEGFR1 genetic variants (rs2296188, rs9943922, rs2296283, rs7324510, rs9554322, rs9582036, rs9554320) were genotyped by KASP. Serum levels of sVEGFR-1 were up-regulated in SLE and positively correlated with disease activity. Furthermore, serum sVEGFR-1 presented a distinctive elevation in SLE in comparison with other rheumatic diseases. Frequencies of allele T of rs2296283 and allele G of rs9554322 were significant lower in SLE patients (*P* = 0.003, *P* = 0.004). Frequencies of genotypes TT of rs2296188 and rs2296283 were declined in patients compared with healthy controls (*P* = 0.039, *P* = 0.033). CC genotype of rs7324510 and rs9582036 was negatively correlated with SLE risk (OR = 0.538, OR = 0.508). Distribution of GG, GC, GG + GC genotypes of rs9554322 were different between SLE patients and healthy controls (*P* = 0.027, *P* = 0.036, *P* = 0.010). Moreover, frequency of TC genotype of rs7324510 was higher in SLE patients with lupus headache (χ^2^ = 9.924, *P* = 0.039) and frequency of TC genotype of rs9943922 was lower in patients with cylindruriain (χ^2^ = 7.589, *P* = 0.026). Frequencies of allele C of rs7324510 and allele T of rs9943922 were decreased in SLE patients with cylindruria and hypocomplementemia, respectively (χ^2^ = 4.195, *P* = 0.041, χ^2^ = 3.971, *P* = 0.046). However, frequency of allele C of rs9554322 was increased in SLE patients with pyuria (χ^2^ = 11.702, *P* = 0.001). In addition, SLE patients carrying GG, GC, CC genotypes for rs9554322 had higher levels of serum sVEGFR-1. In conclusion, serum sVEGFR-1 was elevated in SLE patients and may be a disease marker. VEGFR1 gene polymorphisms related to risk of SLE in a Chinese Han population.

## Introduction

Systemic lupus erythematosus (SLE) is a chronic autoimmune disease with a heterogeneous organ involvement that hinges on multiple autoantibodies production. Considering the worldwide estimation of incidence of SLE, there was a regional variation of the disease. European countries had a lower incidence of SLE, whereas Asia, Australia and the Americas had higher incidence^1^. Gender difference also exists in SLE. It is known that occurrence of the disorder in women is more frequent than men, with a ratio of approximately 6:1^2^. The aetiology of SLE has remained elusive, but genetic predisposition, environmental triggers and hormonal factors are demonstrated to involve in. A higher concordance rate in monozygotic twins than dizygotic twins and the high sibling recurrence risk ratio both support a strong heritability of SLE^3^. In the past decade, a variety of genome-wide association studies (GWAS) have recognized over 40 SLE susceptibility loci existing in exons and introns^4,5^.

Soluble vascular endothelial growth factor receptor-1 (sVEGFR-1, also named as sFlt-1) is a nature vascular endothelial growth factor receptor (VEGFR) competitor. The human *VEGFR1* gene is located in chromosome 13q12^6,7^. It was originally found to be expressed on vascular endothelial cells^8^, and then discovered expressed on smooth muscle cells^9^, monocytes^10^, trophoblasts^11^, mesangial cells^12^ and osteoblasts^13^. sVEGFR-1 competes with signaling receptors of VEGF (VEGFR-1/VEGFR-2) by capturing their ligands^14^. It has a negative role in angiogenesis after binding to the VEGF. However, sVEGFR-1 interacts with endothelial cells components, showing an angiogenesis effect. Therefore, sVEGFR-1 exists multiple effects on vessel growth progression and may be a new therapeutic target for VEGF-mediated pathological signaling. In systemic sclerosis (SSc) patients, lower serum level of sVEGFR-1 was detected in comparison with healthy controls^15^. In rheumatoid arthritis (RA) patients, expression of sVEGFR-1 was increased, correlating with VEGF concentration^16^. Serum level of sVEGFR-1 in patients with osteoarthritis (OA) was higher compared with that in non-arthritic controls^16^. *VEGFR1* genetic variant associated with RA disease activity^17^. Collectively, sVEGFR-1 plays important roles in inflammatory and autoimmune diseases. To date, relationship of sVEGFR-1 and lupus is limited. What is the expression profile of sVEGFR-1 in lupus, if *VEGFR1* gene polymorphisms relate to SLE risk needs to be discussed.

## Results

### Demographical and clinical characterization of study subjects

The demographical characteristics of the patients with SLE and controls are shown in Table 1. For SLE patients, age was 38 (27.1–48.4) years. The age was 36 (29.0–40.0) years for RA patients, 42 (39.0–44.0) years for OA patients, 38 (26.50–46.75) years for gout patients, 44 (44.0–46.7) years for Sjögren’s syndrome (SS) patients, 37 (32.0–45.5) years for ankylosing spondylitis (AS) patients (Table 1). The Age of SLE group was matched among all control groups. For gender information about SLE group and controls, there was no gender difference between SLE patients and other study group excepting gout (*P* < 0.001) and AS (*P* < 0.001). In addition, the hypocomplementemia, proteinuria, arthritis and rash were the four dominant clinical characteristics in SLE patients (the proportion of 51.57%, 45.28%, 41.73% and 40.15%, respectively). Other clinical information was contained in Table 1.

**Table 1:** Main demographic and clinical characteristics in patients with SLE and control groups.

Characteristics	SLE	HC	RA	OA	gout	SS	AS
Female (%)/male (%)	89.37/10.06	91.79/8.21	90.00/10.00	86.96/13.04	3.58/96.42	85.19/14.81	17.14/82.86
Age (years)	38 (27.1–48.4)	38 (31.3–47.2)	36 (29.0–40.0)	42 (39.0–44.0)	38 (26.50–46.75)	44 (44.0–46.7)	37 (32.0–45.5)
Lupus headache, n (%)	16 (6.29)	–	–	–	–	–	–
Vasculitis, n (%)	18 (7.08)	–	–	–	–	–	–
Arthritis, n (%)	106 (41.73)	–	–	–	–	–	–
Myositis, n (%)	12 (4.72)	–	–	–	–	–	–
Rash, n (%)	102 (40.15)	–	–	–	–	–	–
Alopecia, n (%)	64 (25.19)	–	–	–	–	–	–
Oral ulcer, n (%)	28 (11.02)	–	–	–	–	–	–
Pleuritis, n (%)	23 (9.06)	–	–	–	–	–	–
Pericarditis, n (%)	22 (8.66)	–	–	–	–	–	–
Fever, n (%)	45 (17.71)	–	–	–	–	–	–
Hypocomplementemia, n (%)	131 (51.57)	–	–	–	–	–	–
ds-DNA, n (%)	60 (23.62)	–	–	–	–	–	–
Thrombocytopenia, n (%)	34 (13.39)	–	–	–	–	–	–
Reduced leukocyte, n (%)	26 (10.24)	–	–	–	–	–	–
Cylindruria, n (%)	12 (4.72)	–	–	–	–	–	–
Hematuria, n (%)	85 (33.46)	–	–	–	–	–	–
Proteinuria, n (%)	115 (45.28)	–	–	–	–	–	–
Pyuria, n (%)	22 (8.66)	–	–	–	–	–	–

*SLE* systemic lupus erythematosus, *HC* healthy controls, *RA* rheumatoid arthritis, *OA* osteoarthritis, *SS* Sjögren’s syndrome, *AS* ankylosing spondylitis.

### Correlation between serum levels of sVEGFR-1 and SLE

**Figure 1:**
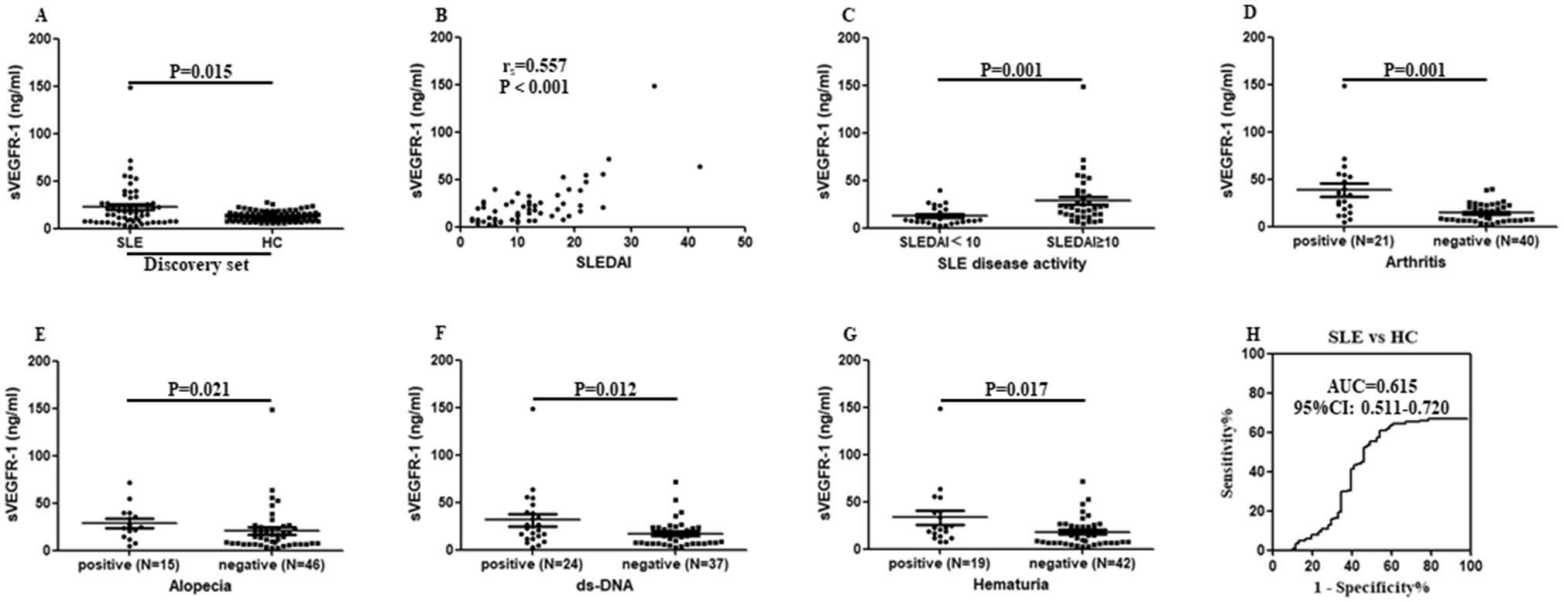
Serum levels of sVEGFR-1 in the discovery set. (**A**) Enzyme-linked immunosorbent assay was used to detect serum sVEGFR-1 levels in 61 systemic lupus erythematosus (SLE) patients and 94 healthy controls. Every symbol expresses an independent SLE patient and healthy control. (**B**) Correlation between SLEDAI and serum levels of sVEGFR-1. (**C**) Difference of serum levels of sVEGFR-1in SLE patients with less-active period and active period. (**D**–**G**) Difference of serum levels of sVEGFR-1 in several characteristics of SLE patients. (**H**) Potential of sVEGFR-1 as a disease marker for SLE, analyzed by receiver operating characteristic curve analysis.

**Figure 2:**
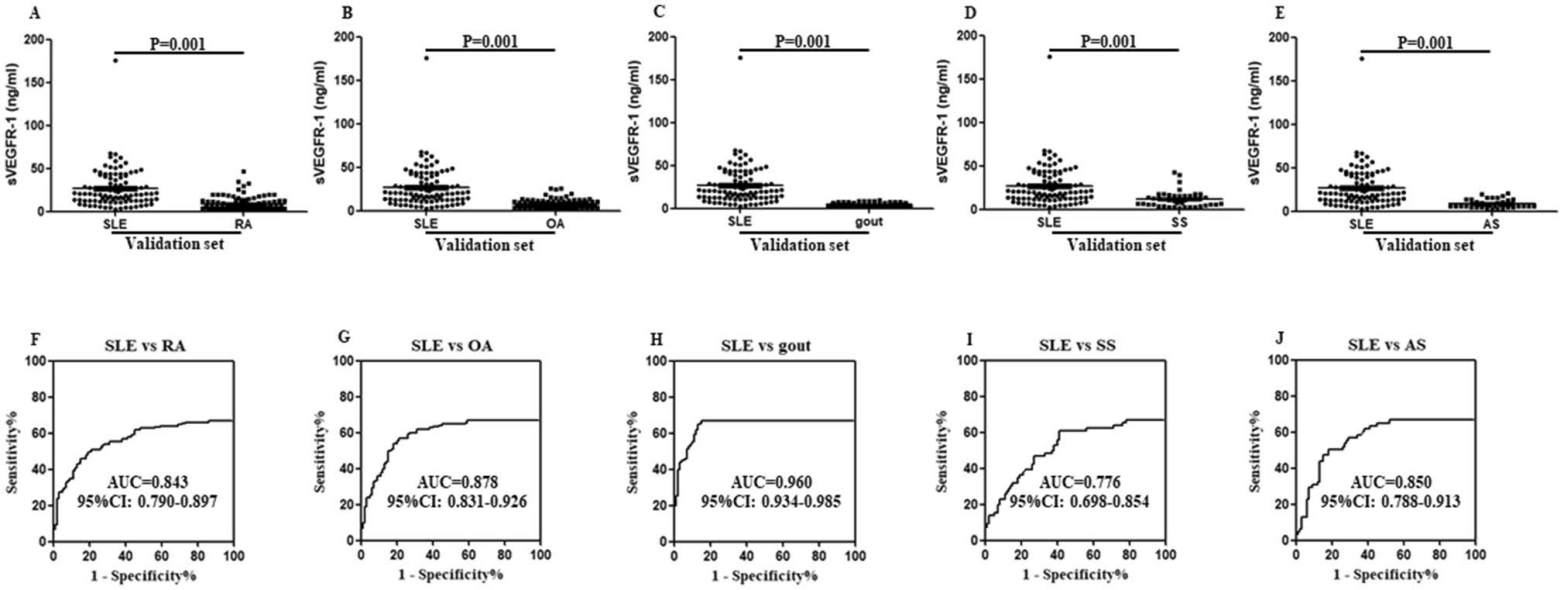
Serum levels of sVEGFR-1 in the validation set. (**A**–**E**) Differences of serum levels of sVEGFR-1 were tested between SLE and other rheumatic diseases (RA, OA, gout, SS and AS). (**F**–**J**) ROC curve analysis was performed to distinguish SLE from other rheumatic diseases.

Concentration of sVEGFR-1 in patients with 61 SLE was higher in comparison with that in 94 healthy subjects (17.738 (7.604–26.286) vs 12.115 (8.655–12.115) ng/ml, *P* = 0.015, Fig. 1A). Correlation between sVEGFR-1 levels and SLE disease activity index (SLEDAI) was calculated, showing a statistically significant correlation (r_s_ = 0.557, *P* < 0.001, Fig. 1B). Higher levels of serum sVEGFR-1 were discovered in active SLE patients than in less-active patients (7.968 (5.325–20.421) vs 22.435 (13.491–36.642) ng/ml, *P* < 0.001, Fig. 1C). When discussing the association of serum levels of sVEGFR-1 and SLE clinical and laboratory characteristics, differences were existed in the patients with arthritis, alopecia, ds-DNA, hematuria (*P* = 0.001, *P* = 0.021, *P* = 0.012, *P* = 0.017, respectively, Fig. 1D–G). The other information about the relationships between sVEGFR-1 and SLE clinical characteristics were exhibited in Table 2. Moreover, ROC curve showed that the AUC was 0.615 (95% CI 0.511–0.720) (Fig. 1H). These results suggested that serum sVEGFR-1 was up-regulated in SLE and positively correlated with disease activity. In validation set, 100 SLE patients and 385 disease controls (including RA, OA, gout, SS and AS) were compared to evaluate difference of serum levels of sVEGFR-1. Analysis indicated that serum concentration of sVEGFR-1 was significantly higher in SLE than that in RA, OA, gout, SS and AS groups (20.987 (13.080–40.885) vs 6.151 (3.614–12.134), 6.208 (4.129–10.293), 3.862 (2.790–5.817), 10.975 (5.513–15.963), 7.851 (5.268–7.851) ng/ml, all *P* < 0.001, Fig. 2A–E). Serum sVEGFR-1 in SLE patients compared with that in RA, OA, gout, SS and AS patients showed AUC of 0.843, 0.878, 0.960, 0.776, 0.850, respectively (Fig. 2F–J). Thus, these results revealed that serum sVEGFR-1 presented a distinctive elevation in SLE.

**Table 2:** Analysis of serum sVEGFR-1 levels in SLE by clinical features.

Clinical features	Positive n (%)	Negative n (%)	Z	*P*
Vasculitis	10 (16.39)	51 (83.61)	1.013	0.311
Arthritis	21 (34.43)	40 (65.57)	3.931	0.001
Rash	23 (37.70)	38 (62.30)	0.551	0.582
Alopecia	15 (24.59)	46 (75.41)	2.311	0.021
Fever	11 (18.03)	50 (81.97)	1.163	0.245
Hypocomplementemia	21 (34.43)	40 (65.57)	1.199	0.230
ds-DNA	23 (37.70)	38 (62.30)	2.188	0.029
Thrombocytopenia	17 (27.87)	44 (72.13)	0.434	0.664
Hematuria	19 (31.15)	42 (68.85)	2.383	0.017
Proteinuria	31 (50.82)	30 (49.18)	1.774	0.076
SLEDAI	38 (62.30)	23 (37.70)	3.199	0.001

*sVEGFR-1* soluble vascular endothelial growth factor receptor-1, *SLE* systemic lupus erythematosus, *SLEDAI* SLE disease activity index.

### Polymorphisms of *VEGFR1* and risk of SLE

To investigate whether polymorphisms of *VEGFR1 gene* affect SLE, seven SNPs were analyzed between SLE patients and healthy controls. No deviation from the HWE test was observed in patients and controls for each polymorphism (*P* > 0.05, Table 3). Results found that SLE associated with genotypes or alleles of following SNPs: rs2296188, rs9943922, rs2296283, rs7324510, rs9554322, rs9582036. The genotypes and alleles frequencies were summarized in Table 4. Compared with healthy controls, frequency of rs2296283 allele T was strongly declined in SLE patients (OR = 0.710, 95% CI 0.567–0.891, *P* = 0.003). Similarly, frequency of rs9554322 allele G was significantly associated with SLE (OR = 0.667, 95% CI 0.468–0.901, *P* = 0.004). As for frequencies of *VEGFR1* genotypes, the most significant difference was observed in rs2296283, by which the genotype TT was dramatically decreased in SLE patients compared with healthy controls (OR = 0.513, 95% CI 0.327–0.804, *P* = 0.004). There were decreased frequencies of TT + TC in patients with SLE as compared with healthy controls (OR = 0.588, 95% CI 0.394–0.876, *P* = 0.009). Frequencies of all rs9554322 genotypes (GG, GC, GG + GC) were lower in SLE group (OR = 0.417, 95% CI 0.192–0.907, *P* = 0.027; OR = 0.694, 95% CI 0.493–0.977, *P* = 0.036; OR = 0.649, 95% CI 0.468–0.901, *P* = 0.010). For rs2296188 and rs9943922, frequencies of TT were both lower in SLE patients when compared with controls (OR = 0.578, 95% CI 0.344–0.972, *P* = 0.039; OR = 0.619, 95% CI 0.398–0.962, *P* = 0.033). Moreover, we found marginal differences of genotype CC in rs7324510 and rs9582036 in SLE patients compared with healthy controls (OR = 0.538, 95% CI 0.296–0.976, *P* = 0.041; OR = 0.508, 95% CI 0.262–0.907, *P* = 0.046). Frequencies of rs9554320 genotypes, although not statistically significant, were lower in SLE patients as compared with healthy subjects (data not show).

**Table 3:** The Hardy–Weinberg's expectation test in patients and controls of seven SNPs.

	SLE		HC	
rs2296188	χ^2^ = 0.001	*P* = 0.999	χ^2^ = 3.434	*P* = 0.180
rs9943922	χ^2^ = 0.032	*P* = 0.984	χ^2^ = 1.199	*P* = 0.549
rs2296283	χ^2^ = 1.053	*P* = 0.591	χ^2^ = 0.368	*P* = 0.832
rs7324510	χ^2^ = 0.037	*P* = 0.982	χ^2^ = 4.662	*P* = 0.097
rs9554322	χ^2^ = 0.009	*P* = 0.995	χ^2^ = 0.167	*P* = 0.920
rs9582036	χ^2^ = 0.276	*P* = 0.871	χ^2^ = 5.685	*P* = 0.058
rs9554320	χ^2^ = 0.453	*P* = 0.797	χ^2^ = 0.136	*P* = 0.934

*SLE* systemic lupus erythematosus, *HC* healthy controls, *SNPs* single nucleotide polymorphisms.

**Table 4:** Allele and genotype frequencies of seven SNPs in the *VEGFR1* gene in SLE patients and healthy controls.

SNP	Genotype	SLE (N = 254) n (%)	Controls (N = 390) n (%)	OR (95% CI)	*P* value
rs2296188	TT	25 (9.8)	62 (15.9)	0.578 (0.344–0.972)	0.039
	TC	109 (43.0)	156 (40.0)	1.001 (0.714–1.404)	0.993
	TT + TC	134 (52.8)	218 (55.9)	0.881 (0.641–1.211)	0.434
	CC	120 (47.2)	172 (44.1)	Reference	
	T	159 (31.3)	280 (35.9)	0.814 (0.614–1.032)	0.089
	C	349 (68.7)	500 (64.1)	Reference	
rs9943922	TT	62 (24.4)	127 (32.6)	0.619 (0.398–0.962)	0.033
	TC	125 (49.2)	178 (45.6)	0.891 (0.601–1.321)	0.565
	TT + TC	187 (73.6)	305 (78.2)	0.778 (0.538–1.124)	0.181
	CC	67 (26.4)	85 (21.8)	Reference	
	T	249 (49.0)	432 (55.4)	0.775 (0.619–0.969)	0.025
	C	259 (51.0)	348 (44.6)	Reference	
rs2296283	TT	79 (31.1)	154 (39.5)	0.513 (0.327–0.804)	0.004
	TC	115 (45.3)	176 (45.1)	0.654 (0.427–1.003)	0.051
	TT + TC	194 (76.4)	330 (84.6)	0.588 (0.394–0.876)	0.009
	CC	60 (23.6)	60 (15.4)	reference	
	T	273 (53.8)	484 (62.1)	0.710 (0.567–0.891)	0.003
	C	235 (46.2)	296 (37.9)	Reference	
rs7324510	CC	17 (6.7)	46 (11.8)	0.538 (0.296–0.976)	0.041
	CA	94 (37.0)	136 (34.9)	1.005 (0.717–1.410)	0.975
	CC + CA	111 (43.7)	182 (46.7)	0.887 (0.646–1.220)	0.460
	AA	143 (56.3)	208 (53.3)	Reference	
	C	128 (25.2)	229 (29.3)	0.810 (0.629–1.044)	0.103
	A	380 (74.8)	551 (70.7)	Reference	
rs9554322	GG	9 (3.5)	28 (7.2)	0.417 (0.192–0.907)	0.027
	GC	77 (30.3)	144 (36.9)	0.694 (0.493–0.977)	0.036
	GG + GC	86 (33.8)	172 (44.1)	0.649 (0.468–0.901)	0.010
	CC	168 (66.2)	218 (55.9)	Reference	
	G	95 (18.7)	200 (25.7)	0.667 (0.507–0.878)	0.004
	C	413 (81.3)	580 (74.3)	Reference	
rs9582036	CC	13 (5.1)	37 (9.4)	0.508 (0.262–0.907)	0.046
	CA	80 (31.5)	120 (30.8)	0.965 (0.682–1.364)	0.840
	CC + CA	93 (36.6)	157 (40.2)	0.857 (0.619–1.188)	0.354
	AA	161 (63.4)	233 (59.8)	Reference	
	C	106 (20.8)	193 (24.8)	0.802 (0.613–1.049)	0.108
	A	402 (79.2)	587 (75.2)	Reference	
rs9554320	CC	141 (55.5)	234 (60.0)	1.019 (0.498–2.088)	0.957
	CA	100 (39.4)	134 (34.3)	1.263 (0.607–2.632)	0.532
	CC + CA	241 (94.9)	368 (94.3)	1.109 (0.548–2.242)	0.775
	AA	13 (5.1)	22 (5.6)	Reference	
	C	382 (75.2)	602 (77.2)	0.931 (0.397–1.869)	0.841
	A	126 (24.8)	178 (22.8)	Reference	

*SNP* single nucleotide polymorphism, *SLE* systemic lupus erythematosus, *OR* odds ratio, *CI* confidence interval.

### *VEGFR1* haplotypes and SLE risk

**Figure 3:**
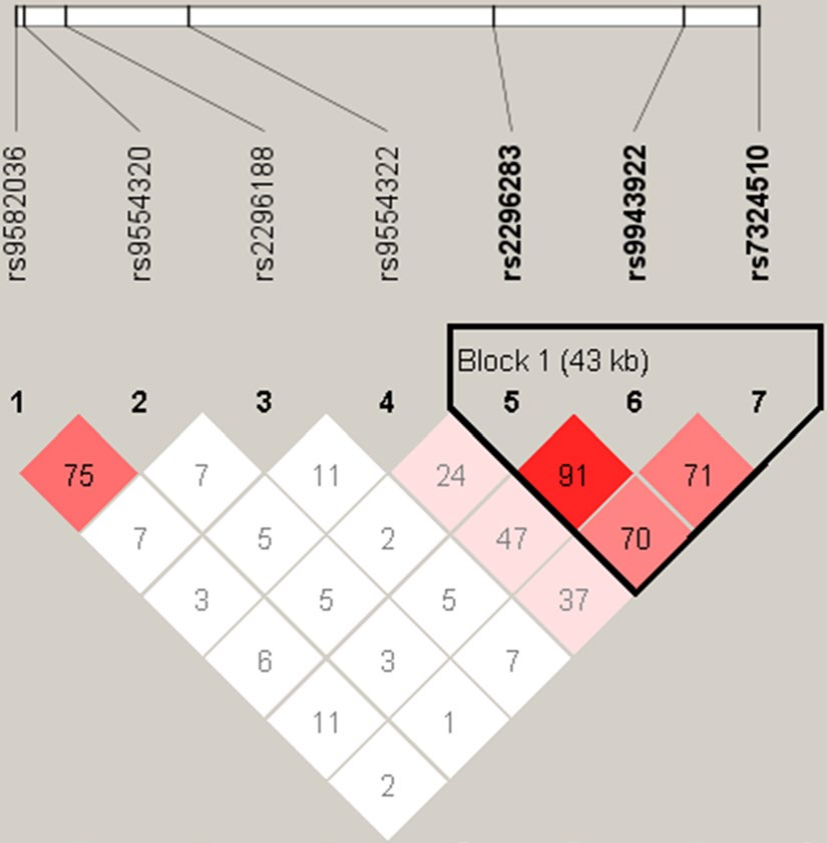
Linkage disequilibrium of seven SNPs. Seven SNPs (rs2296188, rs9943922, rs2296283, rs7324510, rs9554322, rs9582036, rs9554320) were selected in the study. The intensity of LD is reflected in the color and numeric value (D′) of each box. Red and pink represent significant linkage, light blue and white indicate no linkage. D' value varies from 0 to 1, and the 1 value represents the maximum linkage.

In the present study, we exerted the haplotype analysis through constructing a block which comprised rs2296283, rs9943922 and rs7324510 (D′ = 0.915, r^2^ = 0.521; D′ = 0.716, r^2^ = 0.221; D′ = 0.700, r^2^ = 0.131) (Fig. 3). Results revealed that the frequencies of CTA haplotype were higher in SLE patients in comparison with healthy controls (OR = 1.435, 95% CI 1.137–1.810, *P* = 0.002). Proportions of CTC and TTA haplotypes were fewer in SLE patients (OR = 0.234, 95% CI 0.081–0.674, *P* = 0.003; OR = 0.303, 95% CI 0.197–0.465, *P* = 0.001). The other haplotypes were not figured out statistical significance (Table 5).

**Table 5:** Haplotype analysis between SLE patients and Healthy controls.

Haplotype	SLE n (%)	Controls n (%)	χ2	*P* values	OR (95% CI)
CTA	217.23 (42.8)	266.00 (34.1)	9.320	0.002	1.435 (1.137–1.810)
CTC	4.03 (0.8)	25.54 (3.3)	8.557	0.003	0.234 (0.081–0.674)
TCA	125.43 (24.7)	164.34 (21.1)	2.101	0.147	1.218 (0.933–1.589)
TCC	119.83 (23.6)	173.21 (22.2)	0.252	0.615	1.071 (0.820–1.398)
TTA	27.74 (5.5)	123.62 (15.8)	32.701	0.001	0.303 (0.197–0.465)
TTC	0.00 (0.0)	20.84 (2.7)	–	–	–
CCA	9.61 (1.9)	0.04 (0.0)	–	–	–
CCC	4.13 (0.8)	6.41 (0.8)	–	–	–

Haplotype comprised rs2296283, rs9943922 and rs7324510.

*SLE* systemic lupus erythematosus, *OR* odds ratio, *CI* confidence interval.

### Association of *VEGFR1* gene polymorphisms with clinical and laboratory features in SLE

Given that SLE patients had diverse phenotypes and genetic predisposition, we explored association between *VEGFR1* polymorphisms and clinical, laboratory features. The genotype and allele frequencies of *VEGFR1* polymorphisms in SLE patients with different clinical manifestations were listed in Table 6. As the results shown, there was an increased frequency of TC genotype in patients with lupus headache in contrast to patients without this feature for rs7324510 (χ^2^ = 9.924, *P* = 0.039). For rs9943922, TC genotype was different in patients with cylindruria as compared with patients without this feature (χ^2^ = 7.589, *P* = 0.026). Distribution of CC, CA, AA genotypes of rs9582036 and rs9554320 was different between SLE patients with and without pyuria (χ^2^ = 14.437, *P* = 0.003; χ^2^ = 15.074, *P* = 0.001). In addition, C allele frequency of rs7324510 was lower in SLE patients with hypocomplementemia comparing to those without (χ^2^ = 4.195, *P* = 0.041). T allele frequency of rs9943922 polymorphism was different between SLE patients with cylindruria and those without (χ^2^ = 3.971, *P* = 0.046). It was found that the C allele frequency of rs9554322 was increased in SLE patients with pyuria (χ^2^ = 11.702, *P* = 0.001). No significant association was found between the other SNPs and clinical, laboratory manifestations of SLE (Supplementary Table 1).

**Table 6:** Analysis of *VEGFR1* gene polymorphisms in SLE by clinical, laboratory features.

Clinical features	rs9943922							rs7324510						
	Genotype frequency, n (%)			*P* value	Allele frequency, n (%)		*P* value	Genotype frequency, n (%)			*P* value	Allele frequency, n (%)		*P* value
	TT	TC	CC		T	C		CC	CA	AA		C	A	
**Lupus headache**														
Positive	2 (12.50)	6 (37.50)	8 (50.00)	0.099	11 (34.38)	21 (65.62)	0.087	4 (25.00)	6 (37.50)	6 (37.50)	0.039	12 (37.50)	20 (62.50)	0.098
Negative	60 (25.21)	119 (50.00)	59 (24.79)		238 (50.00)	238 (50.00)		13 (5.46)	88 (36.98)	137 (57.56)		116 (24.37)	360 (75.63)	
**Hypocomplementemia**														
Positive	31 (23.66)	65 (49.62)	35 (27.18)	0.960	128 (48.86)	134 (51.14)	0.940	5 (3.82)	46 (38.12)	80 (61.06)	0.095	56 (21.37)	206 (78.63)	0.041
Negative	31 (25.20)	60 (48.78)	32 (26.02)		121 (49.19)	125 (50.81)		12 (9.76)	48 (39.02)	63 (51.22)		72 (29.27)	174 (70.73)	
**Cylindruria**														
Positive	3 (25.00)	2 (16.67)	7 (58.33)	0.026	7 (21.17)	17 (70.83)	0.046	1 (8.33)	3 (25.00)	8 (66.67)	0.676	5 (20.83)	19 (79.17)	0.614
Negative	59 (24.38)	123 (50.83)	60 (24.79)		242 (50.00)	242 (50.00)		16 (6.61)	91 (37.60)	135 (55.79)		123 (25.41)	361 (74.57)	
**Pyuria**														
Positive	2 (9.09)	13 (59.09)	7 (31.82)	0.216	17 (0.386)	27 (61.36)	0.150	1 (4.54)	7 (31.82)	14 (63.64)	0.744	9 (20.45)	35 (79.55)	0.448
Negative	60 (25.86)	112 (48.28)	60 (25.86)		232 (50.00)	232 (50.00)		16 (6.90)	87 (37.50)	129 (55.60)		119 (25.65)	345 (74.35)	

Clinical features	rs9582036							rs9554320						
	Genotype frequency, n (%)			*P* value	Allele frequency, n (%)		*P* value	Genotype frequency, n (%)			*P* value	Allele frequency, n (%)		*P* value
	CC	CA	AA		C	A		CC	CA	AA		C	A	
**Lupus headache**														
Positive	1 (6.25)	4 (25.00)	11 (68.75)	0.840	6 (18.75)	26 (81.25)	0.761	8 (50.00)	7 (43.75)	1 (6.25)	0.896	23 (71.88)	9 (28.13)	0.653
Negative	12 (5.04)	76 (31.93)	150 (63.03)		100 (21.01)	376 (78.99)		133 (55.88)	93 (39.08)	12 (5.04)		359 (75.42)	117 (24.58)	
**Hypocomplementemia**														
Positive	4 (3.05)	44 (33.59)	83 (63.36)	0.269	53 (20.23)	209 (79.77)	0.715	71 (54.20)	54 (41.22)	6 (4.58)	0.790	196 (74.81)	66 (25.19)	0.835
Negative	9 (7.32)	36 (29.27)	78 (63.41)		53 (21.54)	193 (78.46)		70 (56.91)	46 (37.40)	7 (5.69)		186 (75.61)	60 (24.39)	
**Cylindruria**														
Positive	0 (0.00)	6 (50.00)	6 (50.00)	0.306	7 (29.17)	17 (70.83)	0.305	6 (50.00)	6 (50.00)	0 (0.00)	0.584	16 (66.67)	8 (33.33)	0.322
Negative	13 (5.37)	74 (30.58)	155 (64.05)		99 (20.45)	385 (79.55)		135 (55.79)	94 (38.84)	13 (5.37)		366 (75.62)	118 (24.38)	
**Pyuria**														
Positive	4 (18.18)	11 (50.00)	7 (31.82)	0.003	18 (40.91)	26 (59.09)	0.001	5 (22.73)	13 (59.09)	4 (18.18)	0.001	24 (54.54)	20 (45.45)	0.150
Negative	9 (3.88)	69 (29.74)	154 (66.38)		88 (18.97)	376 (81.03)		136 (58.62)	87 (37.50)	9 (3.88)		358 (77.16)	106 (22.84)	

*SLE* systemic lupus erythematosus.

### Variation of *VEGFR1* and concentration of serum sVEGFR-1

**Figure 4:**
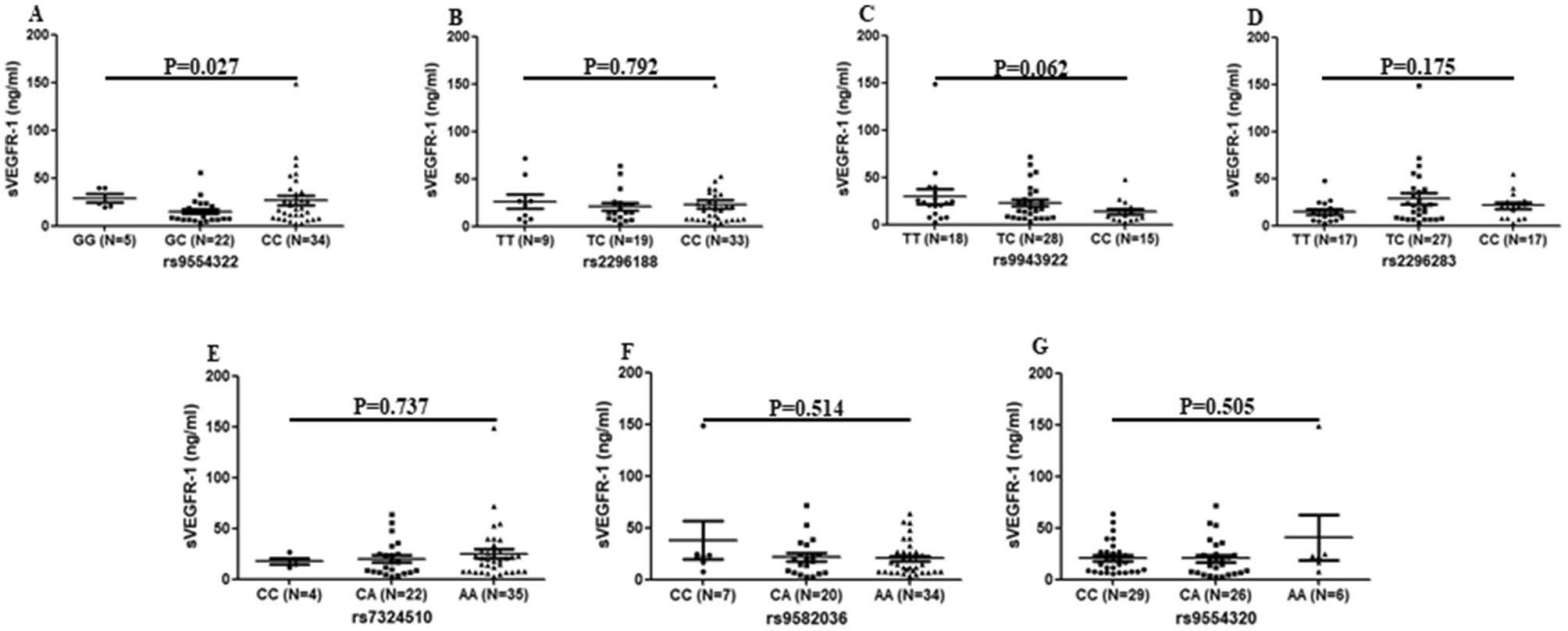
Genotypes of *VEGFR1* gene and serum sVEGFR-1. Serum levels of sVEGFR-1 were examined by ELISA and *sVEGFR1* gene polymorphisms (rs2296188, rs9943922, rs2296283, rs7324510, rs9554322, rs9582036, rs9554320) were genotyped by PCR in SLE patients (N = 61). (**A**) SLE patients carrying GG genotypes (N = 5) of rs9554322 in *VEGFR1* gene showed higher expression of sVEGFR-1. (**B**–**G**) Protein concentrations of sVEGFR-1 were not related to genotypes of rs2296188, rs9943922, rs2296283, rs7324510, rs9582036 and rs9554320 in *VEGFR1* gene. Comparison of sVEGFR-1 values among three groups was performed by the Kruskal–Wallis test.

To discuss possible significance of *VEGFR1* SNPs on serum sVEGFR-1 concentration, serum sVEGFR-1 levels were compared according to genotypes of individual SNP. Analysis indicated that serum levels of sVEGFR-1 were significantly different among SLE patients carrying GG, GC, CC genotypes for rs9554322 (*P* = 0.027, Fig. 4A). The other SNPs were not related to serum levels of sVEGFR-1 (Fig. 4B–G).

### Statistical power

According to α = 0.05, OR = 1.8, and the MAF of each SNP, we calculated the statistical power. In the present study, the following powers were listed: 0.953 for rs2296188, 0.953 for rs9943922, 0.953 for rs2296283, 0.948 for rs7324510, 0.951 for rs9554322, 0.941 for rs9582036, 0.948 for rs9554320, respectively.

## Discussion

In the present study, we revealed that sVEGFR-1 serum levels associated with severity of SLE as well as *VEGFR1* genetic variants existed in Chinese Han population. These findings added evidence that serum sVEGFR-1 and *VEGFR1* gene were implicated in SLE pathogenesis. To our knowledge, this is the first study describing the network of serum sVEGFR-1, *VEGFR1* gene and SLE risk. According to the findings, we observed that SLE patients showed higher serum levels of sVEGFR-1 which positively correlated with disease activity. Furthermore, genotype TT of rs2296188, TT of rs9943922, TT, TT + TC of rs2296283, CC of rs7324510, GG, GC, GG + GC of rs9554322 and CC of rs9582036 related to genetic susceptibility of SLE. SLE patients carrying rs2296283 G allele, rs9554322 T allele had decreased risk of SLE. Moreover, we found that *VEGFR1* rs9554322 polymorphism may be a genetic factor for regulating sVEGFR-1 expression.

SLE is an autoimmune disease with a panel of clinical manifestations such as lupus nephritis that results from a variety of immunological and vascular abnormalities. Relationship between sVEGFR-1 and SLE is not clearly elucidated to date. VEGF is a crucial factor in circulating angiogenesis. It is able to modulate proliferation of endothelial cells, regulate chemotaxis, and capillary hyperpermeability in angiogenesis^18^. Studies indicated that sVEGFR-1 may be involved in SLE by affecting VEGF-mediated activation of angiogenesis. Several studies found that serum concentration of VEGF was elevated in SLE patients^19–21^. Renal glomerular microvasculature is susceptible to local VEGF-A^22^. VEGF mediates the glomerular endothelial cell proliferation and survival in damaged glomerular capillaries^23^. The dysregulation of VEGF is involved in initiation of glomerular injury^24^. Therefore, regulation of VEGF is important in lupus nephritis development. sVEGFR-1 was encoded by a specifically spliced form of VEGFR-1 mRNA. It is comprised of 656 N-terminal residues of the receptor, with a specific 30 amino acid tail at C-terminus. In SLE patients, plasma levels of sVEGFR-1 were found higher in patients with active lupus nephritis^25^. Similarly, in our study, we found that serum sVEGFR-1 was up-regulated in SLE and positively correlated with disease activity. In the inflammatory circumstance, sVEGFR-1 was secreted by activated monocytes^25^. High concentration of sVEGFR-1 antagonized the protective effect of VEGF, aggravating endothelial cell damage^26^. Therefore, it is hypothesized that sVEGFR-1 was up-regulated in the inflammatory circumstance of SLE, which bound to VEGF, leading to endothelial cell damage, further promoting inflammation in SLE.

Concerning *VEGFR1* gene polymorphisms, our findings found that six SNPs (rs2296188, rs9943922, rs2296283, rs7324510, rs9554322, rs9582036) correlated with risk of SLE. It is worthy that rs9554322 and rs9582036 was the first time found to relation with SLE. Genotypes of GG, GC and GG + GC in rs9554322 were significantly lower in patients than in healthy controls, suggesting that rs9554322 polymorphisms may negatively correlate with the risk of SLE in Chinese Han population (OR = 0.417, OR = 0.694, OR = 0.649). Genotype CC of rs9582036 was related to SLE risk (OR = 0.508). It is possible that regulatory, structural or quantitative polymorphisms at the *VEGFR1* locus may affect VEGF signaling pathway and enhance susceptibility to some angiogenic conditions. In our study, rs2296283 located in the functional 3′-UTR region and other three SNPs (rs2296188, rs9943922 and rs7324510) were within the introns. In a Polish study, there was no statistic difference in allele or genotypes frequencies of rs2296283 between RA patients and healthy controls^17^. On the contrary, we obtained a statistical difference in the frequencies of genotype TT and TT + TC for rs2296283 in SLE patients (OR = 0.513, OR = 0.588). Compared with the Polish study, we further observed statistic differences in genotype TT of rs2296188, TT of rs9943922 and CC of rs7324510 (OR = 0.578, OR = 0.619, OR = 0.538), which were encoded in the introns. Therefore, rs2296283 located in the functional region of *VEGFR1* gene may influence the mRNA translation and stability through regulating polyadenylation, miRNA-mRNA and protein-mRNA interactions. However, further study needs to verify the effect of rs2296283 within the functional region. The significance of SNPs located in non-coding region of *VEGFR1* (introns) were not clear. Several studies showed that polymorphisms in non-coding region of *VEGFR1* possibly silenced or enhanced the transcriptional activity of sVEGFR-1^27,28^. Thus, it is postulated that polymorphisms in the non-coding region may implicate in selective splicing of RNA and promote the transcriptional activity of target protein. It is well known that SNP is the predominant pattern in the genomic DNA sequence variation. The majority of SNPs affected the gene expression instead of the protein composition. In agreement with previous studies, our results exhibited that six of seven SNPs (including rs2296188, rs9943922, rs2296283, rs7324510, rs9582036, rs9554320) were not related with sVEGFR-1 levels. Several GWAS studies indicated that although most of SNPs did not possess the direct ability to change the gene expression, these SNPs sometimes served for the functional counterparts which regulated gene expression and protein assemble^29,30^. Thus, we hypothesized that the six SNPs selected in this study may not directly influence sVEGFR-1 concentration in SLE patients. On the other side, serum levels of sVEGFR-1 were different among rs9554322 genotypes (*P* = 0.029). SLE patients with rs9554322 GG genotype had higher sVEGFR-1 protein levels, suggesting that *VEGFR1* rs9554322 G/C genetic variant may contribute to abnormal sVEGFR-1 serum levels. However, what is the exact role of polymorphisms in *VEGFR1* gene, sVEGFR-1 protein expression needs to be discussed and whether the selected SNPs in the current study can affect *VEGFR1* gene, sVEGFR-1 protein expression needs to be conducted by functional study in the future.

There are several limitations in this study. First, the sample size in our study is relatively limited, where cases were recruited from two hospitals. Considering the large number of Chinese Han population, larger scale and multi-center studies are needed in the future. Second, the clear mechanism of sVEGFR-1 involves in SLE pathogenesis needs to discuss.

In summary, the present study showed that serum levels of sVEGFR-1 were elevated in SLE patients, may be a disease marker, and *VEGFR1* gene polymorphisms related to risk of SLE in a Chinese Han population.

## Materials and methods

### Study subjects

Case–control studies were conducted for sVEGFR-1 serum levels and gene polymorphisms. A total of 254 SLE patients, 385 other rheumatic diseases patients and 390 healthy controls were recruited in the present study. Test for serum levels of sVEGFR-1was performed in two stages. The discovery set included 61 SLE and 94 healthy controls, and the validation set comprised another independent 100 SLE and 385 SLE-free disease controls [100 RA, 100 OA, 100 gout, 44 Sjögren’s syndrome (SS) and 41 ankylosing spondylitis (AS)]. The study of gene polymorphisms consisted of 254 SLE and 390 healthy controls. All patients were recruited from the Department of Rheumatology and Immunology, Affiliated Hospital of Southwest Medical University and Affiliated Minda Hospital of Hubei Minzu University, classified by 1997 American College of Rheumatology (ACR) revised criteria for SLE^31^, 1987 ACR revised criteria for RA^32^, Osteoarthritis Criteria Subcommittee of the American Rheumatism Association (ARA) criteria for OA^33^, 1977 ARA criteria for gout^34^, American-European classification criteria for SS^35^ and Modified New York criteria for AS^36^. The SLE disease activity index (SLEDAI) was calculated to evaluate the disease activity of SLE patients^37^. Based on the SLEDAI score, the disease activity of SLE patients were divided into less-active period (SLEDIA < 10) and active period (SLEDIA ≥ 10). Age and sex matched healthy controls were selected from Jiangyang district center for disease control and prevention in Luzhou, having no history of SLE and other inflammatory autoimmune diseases. All the participants were Chinese Han origin. The Medical Ethics Committee of Southwest Medical University approved our study protocol. Written informed consent was obtained from each subject. Blood samples were collected from patients and healthy controls.

### SNP selection

A systemic search for previous literature about *VEGFR1* gene was performed. Based on the 1,000-genome project (https://www.ncbi.nlm.nih.gov/variation/tools/1000genomes/), all candidate SNPs complied with following screening criteria: pairwise tagging of HapMap population with r^2^ ≥ 0.8; a minor allele frequency (MAF) ≥ 5%; Chinese Han Beijing (CHB) ethnicity. At last, seven SNPs including rs2296188, rs9943922, rs2296283, rs7324510, rs9554322, rs9582036, rs9554320 were selected.

### DNA extraction and genotyping analysis

Peripheral blood was collected from ulnar veins in the fasting and clearheaded state. Samples were centrifuged and serum was stored at – 80 °C until analysis. Genomic DNA was extracted utilizing TIANamp Blood DNA kits (TIANGEN, Beijing, China) in line with manufacturer’s instructions. *VEGFR1* genotyping reactions were completed by Gene Company using KASP (Gene Company, Shanghai, China). Information of KASP primers (Primer_AlleleFAM, Primer_AlleleHEX and Primer_Common) was listed in Supplementary Table 2. To prove the reliability of genotyping results, five percent of the whole samples were repeatedly genotyped. Concordance rate of the repeated cases performed 100%, demonstrating that the results were reliable in this study.

### sVEGFR-1 protein measurement

sVEGFR-1 protein levels of SLE patients and control groups were assessed by enzyme‐linked immunosorbent assay (ELISA) kits (Cusabio, Houston, USA) in accordance with the manufacturer’s protocol. All samples were measured in duplicates and plates were read automatically at an absorbance of 450 nm using LT-4000MS reader (Labtech International Ltd, East Sussex, UK). Concentration was calculated on the basis of a linear standard curve. The detection limit was 0.039 ng/ml.

### Statistical analysis

Data was performed by Statistical Package for the Social Sciences (SPSS Inc., Chicago, version 17.0). Categorical data were expressed as frequency and percentage. According to Shapiro–Wilk test, measurement data were expressed by mean ± standard deviation (SD) when it was normally distributed or median (inter-quartile range) when it was not normally distributed. For comparison of genotype and allele distribution between cases and controls, chi-squared or Fisher’s exact test were used as appropriate. Odds ratio (OR) and 95% confidence interval (CI) were analyzed by logistic regression model. Relationship between two variables was evaluated using Spearman’s rank test. Area of receiver operating characteristic (ROC) curve evaluated the specificity and sensitivity of predictive power of sVEGFR-1 in SLE. Hardy–Weinberg equilibrium (HWE) of genotypes in patients and healthy controls was estimated by chi-squared test. HaploView 4.1 software was used to analyze linkage disequilibrium (LD). *VEGFR1* haplotypes were assessed by using online software SHEsis (https://analysis.bio-x.cn). Statistical power was assessed (https://biostat.mc.vanderbilt.edu/wiki/Main/PowerSampleSize). *P* value lower than 0.05 was significant.

## Supplementary information

Supplementary Table 1.

Supplementary Table 2.

## Data Availability

All data supporting the results of this study are available in the article and supplementary information files or are available from the authors upon request.
