# Supplementary material for: Gene polymorphisms and serum levels of sVEGFR-1 in patients with systemic lupus erythematosus
Source: Sci Rep. 2020 Sep 14;10:15031. doi: 10.1038/s41598-020-72020-8 (PMC7490265; doi:10.1038/s41598-020-72020-8)
Supplement: Supplementary file 1 — Supplementary Table 1. [file 41598_2020_72020_MOESM1_ESM.docx]

| Supplementary table 1 Analysis of VEGFR1 gene polymorphisms in SLE by clinical, laboratory features | | | | | | | | | | | | | | | | | | | | | | | |
| --- | --- | --- | --- | --- | --- | --- | --- | --- | --- | --- | --- | --- | --- | --- | --- | --- | --- | --- | --- | --- | --- | --- | --- |
|  | rs2296188 | | | | | | |  | rs2296283 | | | | | | |  | rs9554322 | | | | | | |
|  | Genotype frequency (n) | | | P value | Allele frequency (n) | | P value |  | Genotype frequency (n) | | | P value | Allele frequency (n) | | P value |  | Genotype frequency (n) | | | P value | Allele frequency (n) | | P value |
| Clinical features | TT | TC | CC |  | T | C |  |  | TT | TC | CC |  | T | C |  |  | GG | GC | CC |  | G | C |  |
| Vasculitis |  |  |  |  |  |  |  |  |  |  |  |  |  |  |  |  |  |  |  |  |  |  |  |
| Positive | 4 | 7 | 7 | 0.265 | 13 | 23 | 0.518 |  | 6 | 10 | 2 | 0.362 | 24 | 12 | 0.107 |  | 14 | 4 | 0 | 0.340 | 33 | 3 | 0.152 |
| Negative | 21 | 102 | 113 |  | 146 | 326 |  |  | 73 | 105 | 58 |  | 249 | 223 |  |  | 154 | 73 | 9 |  | 380 | 92 |  |
| Arthritis |  |  |  |  |  |  |  |  |  |  |  |  |  |  |  |  |  |  |  |  |  |  |  |
| Positive | 11 | 52 | 45 | 0.292 | 74 | 142 | 0.216 |  | 37 | 46 | 25 | 0.632 | 118 | 98 | 0.729 |  | 72 | 30 | 6 | 0.281 | 173 | 43 | 0.549 |
| Negative | 14 | 57 | 75 |  | 85 | 207 |  |  | 42 | 69 | 35 |  | 155 | 137 |  |  | 96 | 47 | 3 |  | 240 | 52 |  |
| Myositis |  |  |  |  |  |  |  |  |  |  |  |  |  |  |  |  |  |  |  |  |  |  |  |
| Positive | 1 | 5 | 6 | 0.973 | 7 | 17 | 0.817 |  | 2 | 7 | 3 | 0.483 | 11 | 13 | 0.426 |  | 8 | 3 | 1 | 0.627 | 19 | 5 | 0.784 |
| Negative | 24 | 104 | 114 |  | 152 | 332 |  |  | 77 | 108 | 57 |  | 262 | 222 |  |  | 160 | 74 | 8 |  | 394 | 90 |  |
| Rash |  |  |  |  |  |  |  |  |  |  |  |  |  |  |  |  |  |  |  |  |  |  |  |
| Positive | 10 | 44 | 48 | 0.998 | 63 | 141 | 0.868 |  | 35 | 45 | 22 | 0.632 | 116 | 88 | 0.248 |  | 63 | 34 | 5 | 0.392 | 162 | 42 | 0.371 |
| Negative | 15 | 65 | 72 |  | 96 | 208 |  |  | 44 | 70 | 38 |  | 257 | 147 |  |  | 105 | 43 | 4 |  | 251 | 53 |  |
| Alopecia |  |  |  |  |  |  |  |  |  |  |  |  |  |  |  |  |  |  |  |  |  |  |  |
| Positive | 9 | 28 | 27 | 0.363 | 46 | 82 | 0.191 |  | 20 | 29 | 15 | 0.999 | 69 | 59 | 0.965 |  | 43 | 19 | 2 | 0.967 | 106 | 22 | 0.612 |
| Negative | 16 | 81 | 93 |  | 113 | 267 |  |  | 59 | 86 | 45 |  | 204 | 176 |  |  | 125 | 58 | 7 |  | 307 | 73 |  |
| Oral ulcer |  |  |  |  |  |  |  |  |  |  |  |  |  |  |  |  |  |  |  |  |  |  |  |
| Positive | 3 | 10 | 15 | 0.711 | 15 | 41 | 0.440 |  | 10 | 10 | 8 | 0.555 | 31 | 25 | 0.797 |  | 19 | 7 | 2 | 0.482 | 47 | 9 | 0.593 |
| Negative | 22 | 99 | 105 |  | 144 | 308 |  |  | 69 | 105 | 52 |  | 242 | 210 |  |  | 149 | 70 | 7 |  | 366 | 86 |  |
| Pleuritis |  |  |  |  |  |  |  |  |  |  |  |  |  |  |  |  |  |  |  |  |  |  |  |
| Positive | 2 | 12 | 9 | 0.643 | 16 | 30 | 0.593 |  | 9 | 9 | 5 | 0.679 | 27 | 19 | 0.480 |  | 18 | 4 | 1 | 0.368 | 40 | 6 | 0.302 |
| Negative | 23 | 97 | 111 |  | 143 | 319 |  |  | 70 | 106 | 55 |  | 246 | 216 |  |  | 150 | 73 | 8 |  | 373 | 89 |  |
| Pericarditis |  |  |  |  |  |  |  |  |  |  |  |  |  |  |  |  |  |  |  |  |  |  |  |
| Positive | 2 | 11 | 9 | 0.780 | 15 | 29 | 0.676 |  | 8 | 10 | 4 | 0.772 | 26 | 18 | 0.456 |  | 16 | 4 | 2 | 0.181 | 36 | 8 | 0.926 |
| Negative | 23 | 098 | 111 |  | 144 | 320 |  |  | 71 | 105 | 56 |  | 247 | 217 |  |  | 152 | 73 | 7 |  | 377 | 87 |  |
| Fever |  |  |  |  |  |  |  |  |  |  |  |  |  |  |  |  |  |  |  |  |  |  |  |
| Positive | 3 | 22 | 20 | 0.563 | 28 | 62 | 0.966 |  | 18 | 18 | 9 | 0.362 | 54 | 36 | 0.189 |  | 26 | 15 | 4 | 0.123 | 68 | 22 | 0.123 |
| Negative | 22 | 87 | 100 |  | 131 | 287 |  |  | 61 | 97 | 51 |  | 219 | 199 |  |  | 142 | 62 | 5 |  | 345 | 73 |  |
| ds-DNA |  |  |  |  |  |  |  |  |  |  |  |  |  |  |  |  |  |  |  |  |  |  |  |
| Positive | 5 | 27 | 28 | 0.875 | 37 | 83 | 0.900 |  | 22 | 23 | 15 | 0.431 | 65 | 55 | 0.915 |  | 42 | 16 | 2 | 0.764 | 97 | 23 | 0.881 |
| Negative | 20 | 82 | 92 |  | 122 | 266 |  |  | 57 | 92 | 45 |  | 208 | 180 |  |  | 126 | 61 | 7 |  | 316 | 72 |  |
| Thrombocytopenia |  |  |  |  |  |  |  |  |  |  |  |  |  |  |  |  |  |  |  |  |  |  |  |
| Positive | 5 | 18 | 11 | 0.154 | 27 | 41 | 0.108 |  | 9 | 17 | 8 | 0.793 | 37 | 31 | 0.905 |  | 21 | 12 | 1 | 0.792 | 55 | 13 | 0.925 |
| Negative | 20 | 91 | 109 |  | 132 | 308 |  |  | 70 | 98 | 52 |  | 236 | 204 |  |  | 147 | 65 | 8 |  | 358 | 82 |  |
| Reduced leukocyte |  |  |  |  |  |  |  |  |  |  |  |  |  |  |  |  |  |  |  |  |  |  |  |
| Positive | 5 | 6 | 15 | 0.052 | 16 | 36 | 0.931 |  | 10 | 10 | 6 | 0.669 | 30 | 22 | 0.546 |  | 18 | 8 | 0 | 0.370 | 44 | 9 | 0.517 |
| Negative | 20 | 103 | 105 |  | 143 | 313 |  |  | 69 | 105 | 54 |  | 243 | 213 |  |  | 150 | 69 | 9 |  | 369 | 87 |  |
| Hematuria |  |  |  |  |  |  |  |  |  |  |  |  |  |  |  |  |  |  |  |  |  |  |  |
| Positive | 6 | 38 | 41 | 0.569 | 51 | 119 | 0.654 |  | 31 | 32 | 22 | 0.212 | 95 | 75 | 0.492 |  | 58 | 23 | 4 | 0.605 | 141 | 29 | 0.501 |
| Negative | 19 | 71 | 79 |  | 108 | 230 |  |  | 48 | 83 | 38 |  | 178 | 160 |  |  | 110 | 54 | 5 |  | 272 | 66 |  |
| Proteinuria |  |  |  |  |  |  |  |  |  |  |  |  |  |  |  |  |  |  |  |  |  |  |  |
| Positive | 13 | 50 | 52 | 0.721 | 78 | 152 | 0.249 |  | 40 | 50 | 25 | 0.501 | 129 | 101 | 0.335 |  | 79 | 31 | 5 | 0.502 | 188 | 42 | 0.817 |
| Negative | 12 | 59 | 68 |  | 81 | 197 |  |  | 39 | 65 | 35 |  | 144 | 134 |  |  | 89 | 46 | 4 |  | 225 | 53 |  |
| SLE, systemic lupus erythematosus. | | | | | | | | | | | | | | | | | | | | | | | |
