# Supplementary material for: Gene polymorphisms and serum levels of sVEGFR-1 in patients with systemic lupus erythematosus
Source: Sci Rep. 2020 Sep 14;10:15031. doi: 10.1038/s41598-020-72020-8 (PMC7490265; doi:10.1038/s41598-020-72020-8)
Supplement: Supplementary file 2 — Supplementary Table 2. [file 41598_2020_72020_MOESM2_ESM.docx]

| Supplementary table 2 Primer sequence of seven SNPs | | | |
| --- | --- | --- | --- |
| SNPs | Primer_AlleleFAM | Primer_AlleleHEX | Primer_Common |
| rs2296188 | GTAGGTTCTAATCTAAAGGCTGTCC | AGTAGGTTCTAATCTAAAGGCTGTCT | GTCTGCATCTTCTGCTGAATGTCCTT |
| rs9943922 | TTGGAAATACGGTCACATACCTCG | TTGGAAATACGGTCACATACCTCA | GTATGTGTACTTAGGAAGAATGTGATCAAT |
| rs2296283 | AATAATGAGACCCCCGGGCC | GTAATAATGAGACCCCCGGGCT | GCAGCCCCCTCGGCCTGAA |
| rs7324510 | CAGTCATAAACTATTCTCTAGGAGAGT | AGTCATAAACTATTCTCTAGGAGAGG | CCAGAAAATATTGCTACAAGCCATAGCAT |
| rs9554322 | CCATGGCCATCTTGTATAAGCAC | CCATGGCCATCTTGTATAAGCAG | CCCCACTGTTGGCCCATATGTAATA |
| rs9582036 | TTACTCCTAAATACTTTAGTATACATTTTGT | TACTCCTAAATACTTTAGTATACATTTTGG | TGTGCCCAGCAACAATAGCCTTCTT |
| rs9554320 | GTAACAGCGGCTTTGCAGTGCT | AACAGCGGCTTTGCAGTGCG | CAAGGTTCCTGTGTGTAGCTGATCAT |
| SNP, single nucleotide polymorphism | | | |
